# A dynamically coupled allosteric network underlies binding cooperativity in Src kinase

**DOI:** 10.1038/ncomms6939

**Published:** 2015-01-20

**Authors:** Zachariah H. Foda, Yibing Shan, Eric T. Kim, David E. Shaw, Markus A. Seeliger

**Affiliations:** 1 Department of Pharmacological Sciences, Stony Brook University, Stony Brook, New York 11794, USA; 2 D. E. Shaw Research, New York, New York 10036, USA; 3 Department of Biochemistry and Molecular Biophysics, Center for Computational Biology and Bioinformatics, Columbia University, New York, New York 10032, USA

## Abstract

Protein tyrosine kinases are attractive drug targets because many human diseases are associated with the deregulation of kinase activity. However, how the catalytic kinase domain integrates different signals and switches from an active to an inactive conformation remains incompletely understood. Here we identify an allosteric network of dynamically coupled amino acids in Src kinase that connects regulatory sites to the ATP- and substrate-binding sites. Surprisingly, reactants (ATP and peptide substrates) bind with negative cooperativity to Src kinase while products (ADP and phosphopeptide) bind with positive cooperativity. We confirm the molecular details of the signal relay through the allosteric network by biochemical studies. Experiments on two additional protein tyrosine kinases indicate that the allosteric network may be largely conserved among these enzymes. Our work provides new insights into the regulation of protein tyrosine kinases and establishes a potential conduit by which resistance mutations to ATP-competitive kinase inhibitors can affect their activity.

## Abstract (web summary)

Protein tyrosine kinases are subject to multiple regulatory mechanisms. Foda *et al*. show that reactants and products of the tyrosine kinase Src bind its catalytic domain with opposite cooperativity, and identify an allosteric network of dynamically coupled amino acids that underlie this behaviour.

## Graphical abstract

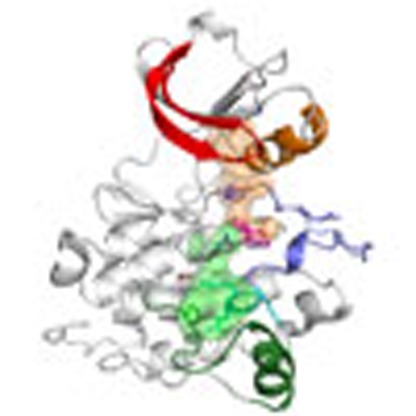

Protein kinases are signalling enzymes that control many vital cellular processes ranging from metabolism to cell division^1^. The biological importance of protein kinases is reflected by the fact that the genes encoding the 518 human protein kinases^2^ constitute ~2% of the human genome. Of these protein kinases, 90 are protein tyrosine kinases (PTKs), which are particularly important in cellular signal transduction. It is thus not surprising that the activity of PTKs is closely regulated^3^ and that dysregulation of their activity underlies many diseases, including schizophrenia^3^, diabetes^4^ and various forms of cancer^5^.

As the core of a highly modular enzyme, the structurally conserved catalytic (kinase) domain of a PTK integrates signals from regulatory domains (for example, SH3, SH2 and PH domains^6^), activators (for example, the activator kinase in an active epidermal growth factor receptor kinase dimer^7^) and post-translational modifications (for example, myristoylation and phosphorylation^8^) within the kinase domain. To regulate the catalytic activity, such signals need to propagate from the protein’s regulatory sites to the ATP- and substrate-binding sites within the kinase domain. Although such communication has been explored previously^9^,^10^, the underlying mechanisms—and whether they lead to an allosteric interaction between the two binding sites—have not been completely elucidated, despite their importance for a better understanding of PTK regulation at a molecular level.

Here we report a highly concerted conformational change observed in molecular dynamics (MD) simulations of the kinase domain of an extensively studied PTK, Src kinase. This change suggests that a dynamically coupled network of amino acids gives rise to cooperativity between ATP and substrate binding. Supporting the findings of the simulations, our biochemical experiments show negative cooperativity between ATP and substrate binding in Src kinase. The proposed allosteric network is further substantiated by our biochemical characterization of the effects of mutations at various residues in the network.

In recent years, important progress has been made in using MD simulations to characterize the dynamics and the intermediate conformations involved in the transitions of a protein kinase domain between its active and inactive states^11^,^12^,^13^. In our MD simulations of Src kinase domain, we observed spontaneous transitions to an inactive conformation highly consistent with that captured in Src crystal structures. Notably, the transition at helix αC in the N-lobe of the kinase is accompanied by concerted conformational changes spanning more than 40 Å across the kinase domain; in addition to helix αC and parts of the catalytic and activation loops, these changes involve the ATP- and substrate-binding sites and the αG helix. These results suggest the existence of an extensive allosteric network in Src kinase. Since this network connects the ATP- and substrate-binding sites, the simulations indicate that binding at these two sites may be cooperative. In our simulations, the concerted conformational changes were induced by the protonation of the aspartate of the Asp-Phe-Gly (DFG) motif at the catalytic site. Previous studies have indicated that ATP binding at the catalytic site is associated with DFG deprotonation^14^, while ADP binding following the phosphoryl transfer leads to protonation of the DFG motif. Our simulations thus suggest that the allosteric network may be switched by the phosphoryl transfer in the kinase catalytic cycle, and that ATP and ADP binding may favour different C-lobe conformational states associated with different substrate-binding characteristics.

The experimental results corroborate the simulations and show negative cooperativity for substrate binding (ATP/peptide) and positive cooperativity for product binding (ADP/phosphopeptide). We find negative cooperativity of substrate binding in Abl and Hck kinases. Bradshaw and colleagues^15^ observe similar behaviour in BTK, suggesting that it may be widely conserved among PTKs. Moreover, our mutagenesis experiments support key atomic details of the allosteric network shown in the simulations. We demonstrate that mutations at distal residues of the identified allosteric network affect substrate binding. The protonation-mimicking D404N mutation at the DFG motif also promotes substrate-peptide binding, providing strong evidence that protonation of the DFG motif plays a central role in this allosteric network. We propose that the allosteric network plays an important role in the enzymatic function of PTKs: release of phosphopeptide promotes ADP release, which is the rate-limiting step of the catalytic cycle. Furthermore, the allosteric network, which contains residues key to the communication between the kinase and the regulatory domains, provides a conduit for regulatory signals to reach the ATP-binding and more distal substrate-binding sites.

## Results

### MD simulations suggest an allosteric network

A previous study of Abl kinase^14^, a member of the PTK family, has shown that the protonation of the DFG aspartate residue significantly perturbs the DFG conformation, and that the DFG conformation may be tightly coupled to the conformation of other parts of the kinase, particularly the αC helix. Analysis of X-ray structures of ADP-bound kinases (for example, PDB entry 1JBP (ref. 16)) and the electrostatic environment of the DFG motif suggests that hydrolysis of the bound ATP may result in the release of the Mg^2+^ between the γ-phosphate of the ATP and the DFG aspartate. This change in the electrostatic environment may raise the pKa of the DFG aspartate and lead to its protonation. Consistent with this notion, our estimate (using the H++ server^17^ based on a continuum model^18^) shows the pKa value to be 4.4 for apo Src kinase, which is raised significantly to 7.5 by the presence of ADP (without Mg^2+^, as in PDB 1JBP). In comparison, the estimated pKa is lowered by the presence of ATP (3.9 with one Mg^2+^ ion or −3.8 with two Mg^2+^ ions). We simulated here both protonated and deprotonated forms of Src kinase domain in the apo state; in addition, we simulated the kinase in its ADP-bound states, with Mg^2+^ ions or with the DFG aspartate protonated. All simulations were initiated from the catalytically active αC-in conformation^19^.

We first simulated Src kinase with the DFG aspartate (Asp404) deprotonated. Six separate simulations from 167 to 375 ns in length (totalling 1,625 ns) suggested that the αC-in conformation of Src kinase remains stable when Asp404 is deprotonated (Supplementary Fig. 1a): in none of the simulations did the αC helix deviate significantly from the αC-in conformation, as indicated by the intact Lys290-Glu310 salt bridge that positions Lys295 for catalysis.

**Figure 1:**
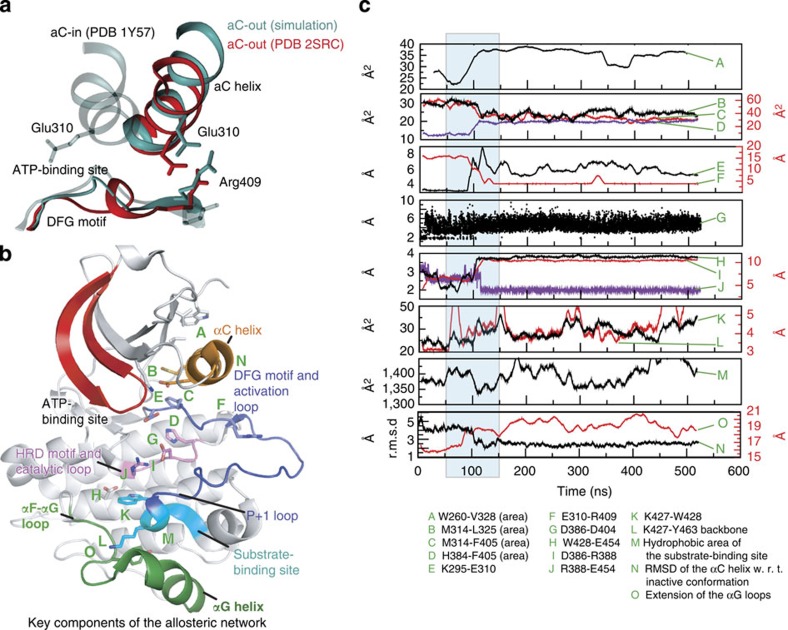
Concerted conformational change simulated in Src kinase up on protonation. (**a**) The movement of the αC helix in the MD simulations. Starting from the active conformation (Simulation—Start), the αC helix (cyan transparent) rotated outwards by about 120° (cyan solid) leading to a salt bridge between Glu310 and Arg409 (Simulation—End). The structure at the end of the simulation resembles that in the crystal structure (red) of autoinhibited Src kinase (Experiment—Inactive, PDB entry 2SRC). (**b**) The location of the contiguous network of residues involved in the concerted conformational change. Green letters denote the approximate location of conformational changes quantified in **c**; a more detailed view of the key residues involved in the conformational change is shown in Fig. 2b,c. (**c**) The structural parameters (contact area, salt bridge or hydrogen bond distance and r.m.s.d.) characterizing the conformational change shown as functions of simulation time. Light blue is used to highlight the narrow time window in which the concerted conformational change occurred.

Protonation of the DFG aspartate, however, enabled the transition to the αC-out conformation: in four out of the eight separate 100-ns-timescale simulations (the eight simulations amounted to a total of 2,400 ns), the kinase departed from the active αC-in conformation, as indicated by the breaking of the Lys290-Glu310 salt bridge (Supplementary Fig. 1a). The protonation produces a conformational change of Asp404 in the DFG motif (with which Phe405 of the motif is coupled) and, in turn, induces the αC-out transition. Without using any crystal structure information about the αC-out conformation, the simulations produced the correct αC-out conformation, in which the helix is ~2 Å backbone root mean squared deviation (r.m.s.d.) from the inactive conformation as captured in PDB entry 2SRC (Fig. 1c, Plot N and Supplementary Fig. 2a, Plot N), and ~8 Å backbone r.m.s.d. away from the initial (active) αC-in conformation.

### Concerted conformational change at substrate-binding sites

**Figure 2:**
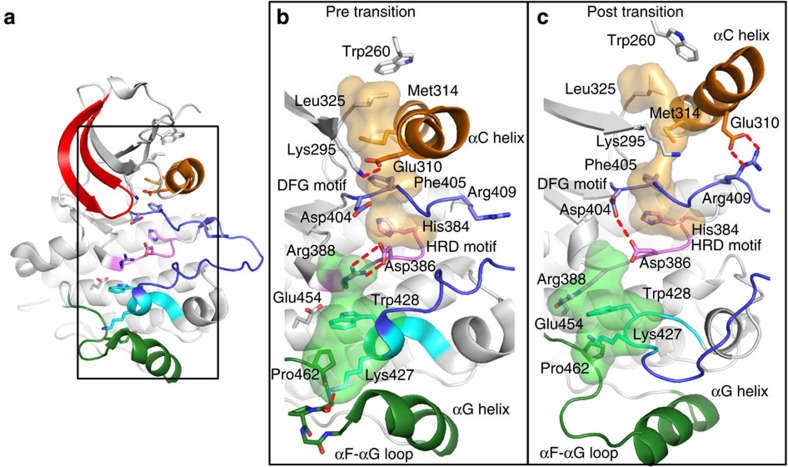
Atomic details of the concerted conformational change. (**a**) An overview of the active Src kinase domain (from PDB entry 1Y57). Helix αC is coloured orange, P-loop red, activation loop blue, catalytic loop pink, P+1 loop cyan and helix αG with αG-αF loop green. Black rectangle indicates the enlarged area to the right. (**b**,**c**) Close-up of the residues identified as part of the allosteric network before (**b**) and after (**c**) the transition is observed in the simulations. The P-loop and parts of the activation loop have been omitted for clarity. The hydrophobic spine residues (orange transparent surface) and the substrate-binding site (green transparent surface) are highlighted. (**b**) Prepared using PDB 1Y57, from which the simulation was initiated. (**c**) Prepared using a snapshot from a simulation, but the key features highlighted (for example, the positions of Trp260 and the Glu310-Arg409 salt bridge) are also seen in the crystal structure of inactive Src kinase (PDB 2SRC). The key hydrogen bond between protonated Asp404 and Asp386 is seen in other kinase crystal structures (for example, PDB 1JOW and 1M14).

A conserved hydrophobic ‘spine’ in the protein kinase domain plays a critical role in maintaining the catalytically active conformation^20^. Consistent with this notion, the αC-out transition was accompanied in our simulations by the breaking of the hydrophobic spine (Leu325, Met314 and Phe405 of the DFG motif, and His384 in Src kinase) in the N-lobe (Figs 1c and 2b). Presumably, the protonation of Asp404 of the DFG motif leads the residue to disengage from Lys295; by the coupling of the DFG residues, this conformational change at Asp404 causes Phe405 to disengage from Met314, breaking the spine and repositioning the αC helix (Fig. 2b). We also observed that Trp260, the first residue of the kinase domain and a key residue in the cross-talk between the regulatory SH2/SH3 and kinase domains in Src^21^,^22^, interacts with Leu325 and thus the hydrophobic spine (Fig. 2b). During the αC-out transition observed in the simulations, Trp260 changed position (Fig. 2b). Given that this repositioning of Trp260 is consistent with the different Trp260 conformations in active and inactive Src kinase structures (this conformation of Trp260 after the αC-out transition has been observed in X-ray structures of inactive Src kinase^23^,^24^) and that it coincides with the αC-out transition in both of the two separate simulations in which the transition is observed (Fig. 1c, Plot A and Supplementary Fig. 2a, Plot A), we conclude that Trp260 is likely conformationally coupled with the hydrophobic spine, and the αC helix.

In addition to the breaking of the hydrophobic spine and the repositioning of Trp260, the αC-out transition was also associated with a conformational change at the part of the ATP-binding site that engages the adenosine moiety of bound nucleotide. It has been shown that the interaction with the adenosine moiety of the bound ATP is the main source of binding affinity in protein kinases^25^. The adenosine group is stabilized by a clamp of two hydrophobic clusters^26^, one from the N-lobe and the other from the C-lobe, which are known as a ‘catalytic spine’^27^. Accompanying the transition to the αC-out conformation, the N-lobe portion of the catalytic spine (Val281, Ala293 and Leu273) and the P-loop are considerably displaced (Supplementary Fig. 3), leading to a disruption of the catalytic spine in addition to the hydrophobic spine, potentially weakening nucleotide binding in the conformation.

The αC-out transition and the other conformational changes in the N-lobe are tightly coupled with conformational changes in the C-lobe and the substrate-binding site (Figs 1c and 2c). After protonation, the DFG aspartate disengages Lys295 and moves towards the catalytic loop in the C-lobe, where it forms a hydrogen bond with Asp386 of the conserved His384/Arg385/Asp386 (HRD) motif. Arg388 in turn breaks away from its salt bridge with Asp386 (Fig. 2b) and engages with Trp428 in a π–cation interaction (Fig. 2c). The effect of the protonation at the DFG motif further propagates from this π–cation interaction, resulting in a repositioning of the P+1 loop in the substrate-binding groove and the αG helix (Figs 1c and 2c). The key to this network of residue–residue interactions is the connection between Asp404 protonation, with the disruption of the Asp386–Arg388 salt bridge, and consequently different packing for Trp428, an anchor residue of the substrate-binding site. To rule out that this simulation observation is merely an artifact of the OPLS-AA force field (see Methods), we repeated the simulations using the AMBER99SB-ILDN force field^28^,^29^,^30^ and obtained essentially the same observations (Supplementary Fig. 4).

The C-lobe conformational change is centred on Lys427 and Trp428, two residues of the P+1 loop. In the active starting conformation (PDB entry 1Y57), the side chain of Trp428 forms a hydrogen bond with Glu454 of the αF helix, while the Lys427 hydrogen bonds to the backbone of the αF–αG loop. The simulations show that, after the transition to the αC-out conformation, these hydrogen bonds are broken and replaced by a tryptophan-centred cation–π–cation interaction^31^ of Arg388, Trp428 and Lys427 (Fig. 2c). Our simulations suggest that he cation–π–cation interaction may be key to the communication between the N- and C-lobes, not only by altering the conformation of the three residues involved, but also by repositioning the αG helix and altering the dynamic properties of the substrate-binding site: The site is much more flexible on protonation of the DFG aspartate residue across the kinase domain (Table 1). The conformational and dynamic coupling of the αC and αG helices, which are spatially separated by more than 40 Å, is particularly noteworthy in light of the fact that they are the two helices in the kinase domain most often involved in kinase regulation^32^.

Notably, the conformational changes across the kinase domain associated with the αC-out transition occurred over a narrow time window and appeared to be highly concerted (Fig. 1c). The near simultaneity of the conformational changes, which was observed in the simulations of both apo and ADP-bound Src kinase (Fig. 1 and Supplementary Fig. 2), suggests a tight coupling between the residues involved in the conformational change. On the basis of this observation, we propose an allosteric network that includes, from the N to the C-lobes: Trp260; the αC helix; the hydrophobic and catalytic spines; the DFG motif of the activation loop; the HRD motif and the conserved Arg388 of the catalytic loop; Lys427 and Trp428 of the P+1 loop; and the αG helix and its adjacent residues (Fig. 1b). This putative allosteric network may allow communication between the ATP- and the substrate-binding sites and allow the control of these sites by the regulatory SH2 and SH3 domains mediated by Trp260. Protonation of the DFG aspartate may switch the network from a conformation favourable for catalysis to a conformation favourable for product release.

### Src, Hck and Abl bind substrates with negative cooperativity

**Figure 3:**
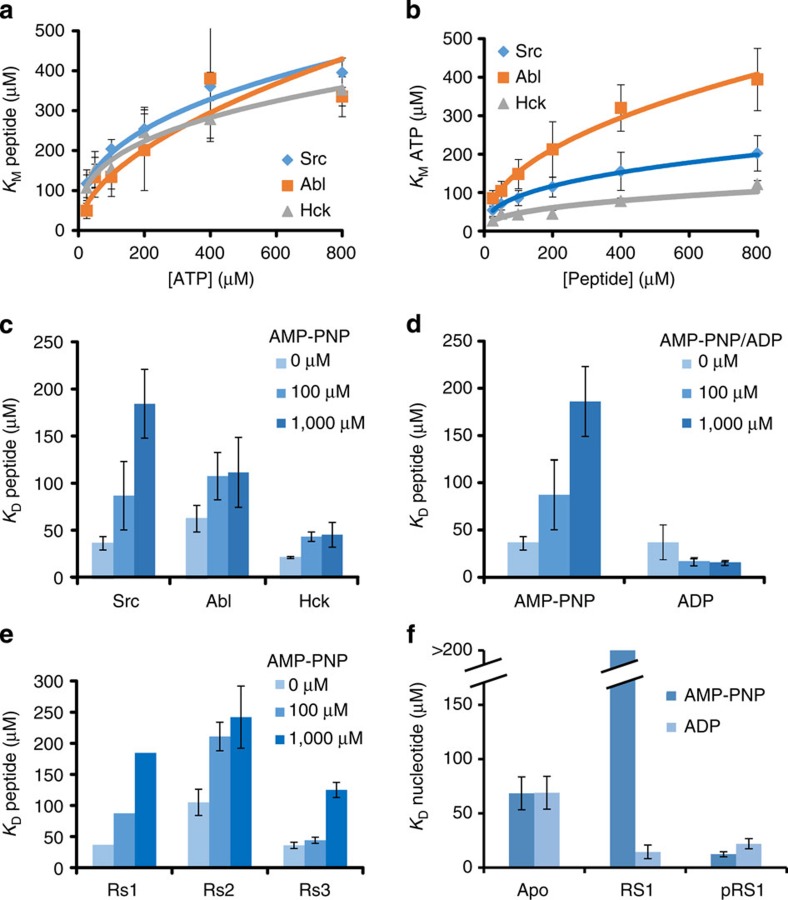
Negative cooperativity of ATP and substrate binding and positive cooperativity of ADP and substrate binding. (**a**) The effect of ATP concentration on substrate *K*_m_ for Src, Abl and Hck kinase domains. (**b**) The effect of substrate peptide concentration on ATP *K*_m_ for Src, Abl and Hck kinase domains. (**c**) The effect of AMP-PNP concentration on substrate *K*_d_ for Src, Abl and Hck kinase domains. (**d**) The effect of ADP and AMP-PNP concentration on substrate *K*_d_ for the Src kinase domain. (**e**) Dissociation constants for peptides of different sequences at increasing AMP-PNP concentration. Rs1: Src-optimal substrate peptide (AEEEIYGEFAKKK); Rs2: peptide sequence (AEEMIYGEFAKKK); Rs3: peptide sequence (GIYWHHY). *K*_m_ values were determined in a kinase activity assay. *K*_d_ values for the substrate peptides were determined using fluorescence anisotropy at 1 μM labelled peptide. *K*_d_ values for AMP-PNP were determined using isothermal titration calorimetry. (**f**) The effect of peptide and phosphorylated peptide on ADP and AMP-PNP binding. All experiments were performed in triplicate and data represent mean values±s.e.m.

The putative allosteric network discussed above may manifest itself in the form of cooperativity between ATP and substrate binding. While positive cooperativity between ATP and substrate binding has been established for the Ser/Thr kinase PKA^33^, such cooperativity has not previously been observed in a tyrosine kinase. Using a fluorescently labelled optimal substrate peptide for Src^34^, we determined a dissociation constant (*K*_d_) of 37±7 (s.e.m.) μM for the interaction of the Src kinase domain with the peptide in the absence of ATP. Higher concentrations of AMP-PNP, a non-hydrolysable ATP analogue, increase the *K*_d_ up to fivefold (Fig. 3c), which indicates negative binding cooperativity. To study the effect of this negative binding cooperativity on kinase activity, we conducted kinase activity assays. We determined the Michaelis–Menten constant (*K*_m_) for substrate peptide and ATP at varying ATP and peptide concentrations, respectively. We found that the *K*_m_ for peptide increases threefold over an ATP concentration range from 50 to 800 μM (Fig. 3a), while the *K*_m_ for ATP increases from 54±22 μM at 50 μM substrate peptide to 202±21 μM in the presence of 800 μM substrate peptide (Fig. 3b).

Src kinase can undergo *trans*-autophosphorylation at Tyr416 in the activation loop, which can affect substrate peptide affinity. We prepared autophosphorylated Src kinase domain and found that it has an increased affinity for substrate peptide (Supplementary Fig. 5). We have also shown previously that Src does not autophosphorylate significantly under the conditions of the enzyme assay^35^. It is thus unlikely that the observed weakening of peptide binding in the presence of ATP is due to Src autophosphorylation. We also demonstrated that negative cooperativity is present in the three-domain construct for Src kinase (Supplementary Fig. 6).

Next we tested whether the sequence of the substrate peptide affected the cooperativity of peptide and ATP binding to the Src kinase domain. We determined the *K*_d_ of different substrate peptides to the Src kinase domain at various concentrations of AMP-PNP (Fig. 3f). The Src-optimal substrate peptide (Rs1 AEEEIYGEFAKKK^34^) contains a negatively charged residue in the P-2 position, important for high affinity, while a variant (Rs2 AEEMIYGEFAKKK) and a short substrate peptide (Rs3 GIYWHHY^36^) are missing this charged residue. We found that all three substrate peptides and AMP-PNP bind to the Src kinase domain with negative cooperativity independent of their overall affinity (Fig. 3f). Since Src kinase follows a random binding mechanism^37^, the observed anti-cooperativity for reactants (ATP and substrate peptide) here cannot be explained by an enzymatic mechanism that follows sequential-ordered substrate binding (for example, in Bruton’s tyrosine kinase^15^). We further show below a positive cooperativity in Src between ATP and phosphopeptide and between ADP and peptide, inconsistent with a sequential-ordered mechanism.

We investigated whether the negative cooperativity for substrate binding is conserved among other Src family kinases and the closely related Abl kinase. We found that the peptide *K*_m_ is, within the margin of error, the same for Src, Hck and Abl, and that it increases with increasing ATP concentration (Fig. 3a). Similarly, the ATP *K*_m_ for the three kinases increases with increasing substrate-peptide concentration (RS1; Fig. 3b). This indicates that the negative cooperativity of substrate binding is shared among multiple tyrosine kinases and may be widely conserved among PTKs.

### Positive cooperativity of product binding to Src kinase

Mg^2+^ ions accompany ATP and ADP binding in protein kinases, mediating interactions between phosphate groups and conserved Asp and Asn residues (Asp404 and Asn391 in Src). On the other hand, in some kinase-ADP co-crystal structures, Mg^2+^ ions are missing. In such cases, the Asp residue of the DFG motif (Asp404 in Src) is almost certainly protonated to prevent negative charge repulsion from the ADP phosphate group only 3.2 Å away (for example, PDB entry 1JBP (ref. 16)). Recent biochemical experiments further showed that ADP release in a kinase catalytic cycle is slower at high Mg^2+^ concentration^38^. We thus infer that the Mg^2+^ ions stabilize ADP binding and their departure occurs before ADP release. Consistent with this notion, our simulations suggest that the presence of Mg^2+^ ions stabilizes ADP binding. In both of the two simulations (of 385 and 225 ns) of ADP-bound Src kinase without Mg^2+^ ions and with Asp404 protonated (a result of the local electrostatic condition), the ADP readily departed from its binding conformation and the protein departed from the αC-in conformation (Supplementary Fig. 1c); in contrast, in the simulation where the ADP is bound with Mg^2+^ ions, the ADP as well as the αC-in conformation remain stable to the end of the significantly longer (over 3,500 ns) simulation (Supplementary Fig. 2d), where the Mg^2+^ ions stabilize ADP binding by mediating the interaction between ADP phosphate groups and Asp404 and Asn391.

We suggest that the allosteric network may switch between the two sets of conformations according to the protonation state of the DFG motif: whereas the binding of ATP-Mg^2+^ leaves the DFG aspartate deprotonated, ADP binding absent of Mg^2+^ ions leads to the protonation of the DFG aspartate. On the basis of these observations, and the negative cooperativity of ATP and substrate-peptide binding, we expect that ADP and substrate peptide will bind with positive cooperativity to the Src kinase domain. We determined the affinity of Src for Src-optimal substrate peptide in the presence of different concentrations of ADP (Fig. 3d). The *K*_d_ of substrate peptide decreases from 37±7 μM in the absence of nucleotide to 17±4 μM at 100 μM ADP and 16±2 μM at 1,000 μM ADP. This is in strong contrast to the effect of AMP-PNP, which increases the *K*_d_ of substrate peptide fivefold over this concentration range. We also showed that the *K*_d_ of ADP is in the same range as for AMP-PNP (45–100 μM) based on isothermal titration calorimetry (Supplementary Fig. 7).

Next we investigated whether the phosphorylation of a peptide alters its binding cooperativity with a nucleotide. We found that phosphorylated Src-optimal substrate peptide (pRs1) reduces the *K*_d_ of AMP-PNP and ADP three- and twofold, respectively (Fig. 3e). Similarly, AMP-PNP leads to ninefold tighter binding of pRs1, while ADP only slightly increased pRs1 binding (Supplementary Fig. 8). To summarize, we find that substrates (ATP and substrate peptide) bind with negative cooperativity to Src kinase, whereas products (ADP and pRS1) and combinations of products and reactants bind with positive cooperativity.

### The DFG aspartate as a key switch in the allosteric network

**Figure 4:**
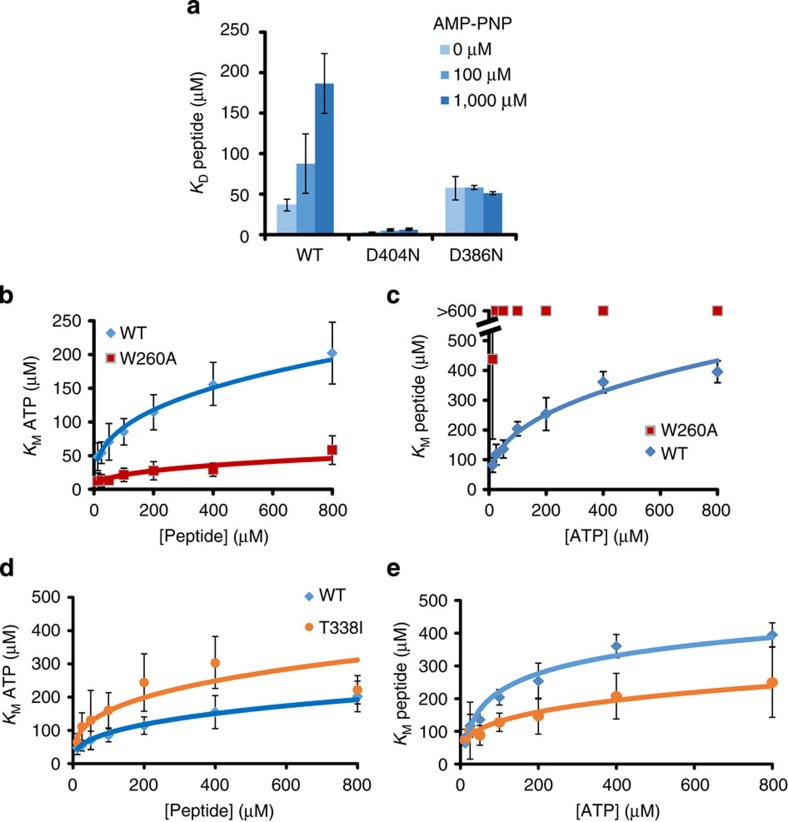
Mutations to the allosteric network producing biochemical phenotypes. (**a**) D404N and D386N mutations leading to stronger substrate binding and disrupting the binding cooperativity, respectively. (**b**) Effect of W260A on ATP-binding affinity. (**c**) Effect of W260A on peptide-binding affinity. (**d**) Effect of T338I on ATP-binding affinity. (**e**) Effect of T338I on substrate-binding affinity. *K*_d_ values were determined using fluorescence anisotropy at 1 μM labelled Src-optimized peptide (Rs1). All experiments were performed in triplicate and data represent mean values±s.e.m.

Our simulations suggest that the protonation of Asp404 of the DFG motif may switch the allosteric network from one conformational state to another. Moreover, our experiments show that ATP binding weakens the substrate binding, while, on the other hand, ADP binding strengthens substrate-peptide binding (Fig. 3d). Since ATP and ADP binding are associated with a deprotonated and protonated DFG aspartate, respectively, we infer that the conformational state that is favoured by DFG protonation is more capable of substrate binding. To examine this possibility, we replaced Asp404 of the DFG motif with asparagine to mimic the protonation of the residue^39^ (Supplementary Fig. 10). We found, as anticipated, that Src D404N binds peptides with 12-fold higher affinity than the wild type (Fig. 4a). The asparagine residue is incapable of chelating Mg^2+^ ions and it thus does not bind ATP favourably (Supplementary Fig. 10c). These findings regarding the D404N mutant further support both the role of the DFG motif as a central conformational switch^14^ and the residue-specific structural mechanism associated with the allosteric network we propose.

We further showed that the negative binding cooperativity between the ATP- and substrate-binding sites requires the presence of Mg^2+^ (Supplementary Fig. 9); this finding is consistent with an important role for Mg^2+^ in maintaining an electrostatic environment in the ATP-binding site that precludes the protonation of the DFG motif. Changing the Mg^2+^ concentration mimics ion binding and release during the catalytic cycle and as such stands in for titrating pH and is consistent with the known pH dependence of drug binding kinetics of Src^40^ and Abl^14^. We also examined the effect of mutations on Asp386, since it may switch between two interactions: it can either share a proton with the protonated DFG Asp404 or form a salt bridge with Arg388 (Fig. 2c). In the D386N mutant, the asparagine can form a hydrogen bond with Asp404 and mimic the former interaction, but not the latter. We thus predict the D386N mutation to disrupt the conformational relay between the αC helix and the substrate-peptide-binding site. As expected, we find that in this mutant the binding cooperativity is disrupted (Fig. 4a).

### Mutation at Trp260 affects substrate binding 40 Å away

To test the reach of the allosteric network into the N-lobe of the kinase domain, we probed Trp260, the residue of the allosteric network that is the most distant from the substrate-binding site. Trp260 is located at the amino (N) terminus of the catalytic domain and is conserved in PTKs. Trp260 may couple the SH2–kinase domain linker conformation to the orientation of the αC-helix^24^, and it thus plays a critical role in mediating interaction of the regulatory SH2/SH3 domains and the Src kinase domain^21^,^22^. The residue is known to be coupled to the ATP-binding site through its interaction with the αC helix^41^ and may be part of the SH2-based activation of Fes kinase^42^. As discussed above, the concerted conformational change at Trp260 that accompanied the αC-out transition in our simulations indicates that the residue is likely integral to the allosteric network in Src kinase.

The mutation of Trp260 to alanine has been shown to enhance the cellular activity of full-length Src kinase, presumably by favouring the enzymatically active conformation^21^. Similarly, in constructs of Hck kinase that include the regulatory SH2 and SH3 domains, the W260A mutant increases kinase activity^22^. On the basis of our simulations and experiments, we predicted that the active-like conformation, stabilized by the W260A mutation, favours ATP binding but weakens substrate binding. Consistent with this model, we found the *K*_m_ of W260A for ATP is two- to fivefold lower than it is in the wild type (Fig. 4b), while the *K*_m_ for substrate peptide is higher than it is in the wild type (Fig. 4c). Given that Trp260 is located more than 40 Å away from the substrate-binding site, these data regarding the W260A mutation are strong evidence for the allosteric network we have proposed.

### Drug resistance mutations and kinase activation

To further examine the proposed conformational and dynamic coupling between the ATP- and substrate-binding sites, we measured the effects of mutation at the ATP-binding site. Thr338, the so-called ‘gatekeeper’ residue, regulates access to a hydrophobic pocket in the ATP-binding site. This mutation is one of the most common clinical resistance mutations to ATP-competitive kinase inhibitors^43^. Intriguingly, the gatekeeper mutation not only results in resistance towards most ATP-competitive kinase inhibitors, but it also activates tyrosine kinases^43^. The crystal structure of Src gatekeeper mutant (T338I) shows that the isoleucine side chain is favourably packed with the hydrophobic spine in the active conformation^43^. On the basis of the crystal structure, we would have expected that the T338I mutation increases the affinity for ATP. Surprisingly, we find that Src T338I exhibits a twofold higher *K*_m_ for ATP (Fig. 4d) than the wild type. Interestingly, at the same time, Src T338I has a twofold lower *K*_m_ for substrate peptide than the wild type at high concentrations of ATP (Fig. 4e). This relationship holds for three-domain Src (Supplementary Fig. 6).

In addition, we tested a number of mutations on residues within or adjacent to the allosteric network, including W428A, E454A and R418P. The ATP and the substrate binding of the mutants we tested are summarized in Supplementary Fig. 10. As shown, these mutants may be roughly classified into three categories: first, those that result in increased ATP affinity and decreased peptide affinity (purple in Supplementary Fig. 10a); second, those that result in decreased affinity for both ATP and peptide substrates (orange in Supplementary Fig. 10a); and third, those that result in increased peptide affinity and decreased ATP affinity (blue in Supplementary Fig. 10a). Notably, we have not identified mutations that lead to an increase in both ATP and substrate binding, which would potentially yield a more potent Src kinase than the evolutionarily selected wild type.

### This allosteric network is likely conserved in other PTKs

The long-range concerted conformational changes observed during the simulated αC-out transition strongly suggest an allosteric network in the catalytic domain of Src kinase. Sequence conservation supports the notion that this network may be shared by many other PTKs, since the key residues (Lys295; Glu310; Arg409; the ‘spine’ residues; the DFG, HRD and RAA/AAR motifs; Trp428 and Lys427 of the P+1 loop; and Glu454 of the αF helix) are highly conserved^44^. In protein Ser/Thr kinases, which bind substrate peptides differently^45^, many of these residues are consistently different from their PTK counterparts. Further supporting the proposed conserved allosteric network, the negative cooperativity of ATP and substrate binding is conserved among Src, Hck and Abl kinases.

## Discussion

**Figure 5:**
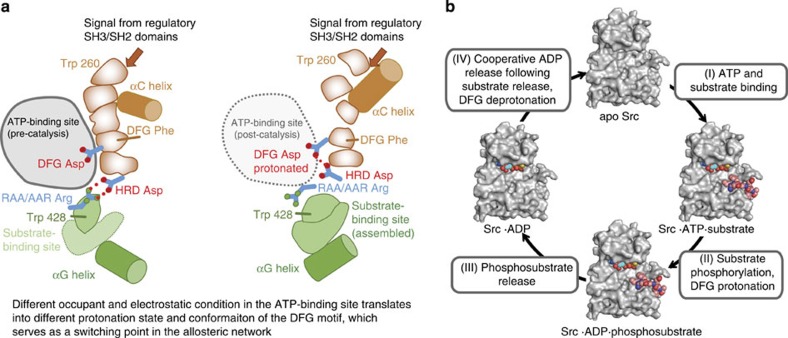
The allosteric network and negative cooperativity in the context of a kinase catalytic cycle. (**a**) The key components of the allosteric network are shown in two configurations. The protonation of the DFG aspartate repositions the Asp and Phe residues, which disrupts the regulatory hydrophobic spine and leads to the αC-out transition and the repositioning of Trp260 in the N-lobe, and in the C-lobe induces the RAA/AAR Arg to form alternative interactions with Trp428 and Glu454, resulting in a rearrangement of the substrate-binding site. (**b**) In a catalytic cycle, the apo kinase (I) binds ATP/Mg^2+^ and substrate, yielding the bisubstrate complex (II), in which the phosphoryl transfer from ATP to substrate occurs. Following the phosphoryl-transfer step, the DFG aspartate becomes protonated (III). The phosphorylated substrate is subsequently released (IV), which weakens ADP binding through the cooperative mechanism and promotes ADP release. The DFG aspartate is then once again deprotonated, and the affinity for ATP increases, starting the catalytic cycle again (I).

Having investigated how a perturbation at the catalytic site (the protonation of the DFG aspartate as a result of local electrostatic changes) may propagate through the Src kinase domain, we propose a dynamically coupled allosteric network connecting the protein’s ATP- and substrate-binding sites (Fig. 5a). The protonation repositions the tightly coupled aspartate and phenylalanine of the DFG motif. In the N-lobe, the repositioning of the phenylalanine disrupts the regulatory hydrophobic spine and leads to the αC-out transition and the repositioning of Trp260. In the C-lobe, the protonated aspartate shares a hydrogen bond with the aspartate of the conserved HRD motif, leading to displacement of the conserved arginine (Arg388) of the RAA/AAR motif, which in turn forms alternative interactions with Trp428 and Glu454, resulting in a rearrangement of the substrate-binding site.

Considering that the allosteric network contains Trp260, which plays a key role in the cross-talk between the kinase domain and the regulatory domains in Src kinase^21^, this network may be coupled with the regulatory SH2 and SH3 domains. That a mutation on this residue (W260A) may weaken the substrate binding in Src kinase by 10-fold suggests that the regulatory signals originating from the SH2 and SH3 domains may reach the substrate-binding site and the adjacent αG helix, far beyond merely reaching the ATP-binding site and the adjacent αC helix as is generally believed^41^. Similarly, allosteric inhibitors (reviewed in ref. 46) target kinases at sites that are adjacent to portions of the proposed allosteric network. Thus, their function may rely on the allosteric network.

This allosteric network facilitates negative binding cooperativity of the substrates (ATP and substrate peptide) and positive binding cooperativity for products (ADP and phosphopeptide) and product/reactant mixtures (ADP/peptide, ATP/phosphopeptide). This has potential effects on kinase activity, kinase substrate specificity and the rise of mutations that cause resistance to ATP-competitive drugs.

Communication between the catalytic- and the substrate-binding sites may promote ADP release, the rate-limiting step of the catalytic cycle (Fig. 5b)^47^. The release of the phosphorylated substrate after the phosphoryl transfer, which is much faster than the rate-limiting step, generally precedes ADP release in a catalytic cycle of a kinase^25^. The positive cooperativity between ADP and phosphopeptide binding implies that the departure of phosphopeptide weakens ADP binding and potentially speeds up the rate-limiting ADP release (Fig. 5b).

Anti-cooperative substrate binding could explain why a subset of resistance mutations to ATP-competitive kinase inhibitors is also activating^43^. Although resistance mutations such as Thr338Ile increase the apparent *K*_m_ for ATP, at the milimolar concentrations of ATP in the cell the mutant kinase is still saturated with this abundant substrate. Concurrently, the affinity of the mutant kinase increases for substrate peptide which in cells is limiting at concentrations below the *K*_m_ (ref. 48).

Consistent with the potential functional importance of the allosteric network, it has been shown that the key DFG motif is not optimized for maximal structural stability. Rather, it is optimized for regulated conformational switches^49^ and moderate energetic difference separates different DFG conformations^50^. The change of electrostatic environment in the ATP-binding site and protonation of the DFG motif may change the sign of the energetic differences^38^. Most likely, the presence of Mg^2+^ ions (and ATP or ADP) in the binding site energetically favours the catalytically active DFG conformation, while the departure of these ions after the completion of the phosphoryl transfer switches the protonation state and the conformation of the DFG motif, and in turn, the allosteric network.

While our discussions focus on the switch between the two conformations, it is clear from existing crystal structures that Src kinase visits other functionally important conformations. A pure two-conformation scenario cannot explain the positive binding cooperativity of phosphopeptide with both ADP and ATP. Further investigations are needed for a comprehensive survey of the conformational spaces of Src and other tyrosine kinases.

In summary, we have used simulations and biochemical experiments to identify a dynamic, long-range allosteric network that facilitates communication between the ATP- and substrate-binding sites. This network of dynamically coupled residues extends the analyses of static protein structures by Taylor and coworkers that led to the description of the regulatory and catalytic spine that stabilizes the active structures of protein kinases. The static and the dynamically coupled spine are not mutually exclusive: the assembled catalytic and regulatory spine is present in the active conformation of Src and Btk kinase. Conversely, the residues that comprise the dynamically coupled spine in Src are also conserved in Btk which like Src shows negative binding cooperativity^51^.

Elucidation of the network’s conformational changes strongly suggests an underlying mechanism for the kinase’s observed negative binding cooperativity. This allosteric network may also mediate communication between kinase regulatory domains and distal parts of the catalytic domain. Moreover, high sequence conservation of the constituent residues suggests that this allosteric network is not isolated to Src, Hck and Abl kinase but is likely common among PTKs.

## Methods

### MD simulations

All simulations reported here were based on the kinase domain from an X-ray structure of the c-Src system in an active conformation (PDB entry 1Y57 (ref. 19)). The side chains incompletely resolved in the PDB entry (Arg469, Glu470, Asp473, Glu476 and Glu488) were constructed using Maestro software (Schrödinger, LLC), which was also used for the determination of the protonation states of residues and the addition of hydrogen atoms. None of the residues other than Asp404 were protonated. The initial placement of the ADP molecule for the simulation of ADP-bound Src was generated by superimposing this Src structure on an ADP-bound structure of cAMP-dependent protein kinase (PDB entry 1JBP (ref. 16)), followed by energy minimization and MD relaxation at low temperature (150 K). The placement of ADP-Mg^2+^ for the simulation of ADP-Mg^2+^-bound Src kinase used another cAMP-dependent protein kinase structure (PDB entry 4IAZ) as template. All simulation systems were set-up by placing the protein at the centre of the simulation box and filling the voided space with water molecules. The dimensions of the simulation boxes were chosen so that no protein atom was within 7.5 Å of the edge. Na^+^ and Cl^−^ ions were added to maintain physiological salinity (150 mM) and to obtain a neutral total charge for the system. All MD simulations, except two, were performed using Desmond^52^ (in the NPT ensemble; 300 K, 1 bar and a Berendsen coupling scheme^53^) with the OPLS-AA/L protein force field^54^,^55^ and SPC water model^56^. The other two (Supplementary Fig. 4) used AMBER99SB-ILDN force field^28^,^29^,^30^ and TIP3P water model^57^,^58^. All bond lengths to hydrogen atoms were constrained using M-SHAKE^59^ as implemented in Desmond^60^. Van der Waals and short-range electrostatic interactions were cutoff at 10 Å. Long-range electrostatic interactions were computed by the Gaussian Split Ewald method^61^ using a 64 × 64 × 64 grid with *σ*=2.357 Å and an on-grid charge-spreading distance *r*_s_=4.19 Å. An r-RESPA integrator^62^ was used with a 2.5-fs time step for all interactions except the long-range electrostatic interactions, which were calculated every 5 fs.

The pKa estimate was performed using the H++ webserver^17^ with atom partial charges defined by the OPLS-AA force field. Qualitatively, the same results were also obtained using the Amber99 force field^29^, which suggests that the results are robust to minor differences in atom partial charges. The coordinates of the protein and ADP atoms are taken from the initial set-up for the simulations.

### Protein purification

Kinase domain constructs of chicken c-Src (residues 251–533), human Hck (residues 166–445) and human c-Abl (residues 229–512) were expressed as previously described^35^,^63^. Mutations were introduced into the chicken c-Src kinase domain by site-directed mutagenesis and verified by DNA sequencing. Protein purity was at least 95% as determined by Coomassie staining.

### Kinase activity assays

For the continuous spectrophotometric assay^64^, 12.5–800 μM ATP Src-optimal substrate peptide (AEEEIYGEFAKKK)^34^ was combined with 12.5–800 μM ATP. Concentrations of Src kinase domain used for these assays were 25 nM. The initial velocities were plotted and fit to the Michaelis–Menten equation in Kaleidagraph (Synergy Software) to determine *K*_m_.

### Anisotropy binding assay

Src kinase domain or mutant was titrated to 1 μM of the N-terminal fluorescein-labelled peptides (Rs1: AEEEIYGEFAKKK; Rs2: AEEMIYGEFAKKK; Rs3: GIYWHHY; RS Synthesis, Louisville) in 100 mM Tris, pH 8.0 and 10 mM MgCl_2_ at 25 °C. After a 1-min equilibration, the increase in the fluorescence anisotropy of the fluorescently labelled ligand was recorded using a Fluoromax 4 (Horiba) and fitted against a quadratic binding equation in Kaleidagraph (Synergy Software) to yield the *K*_d_. Error bars represent the s.e. of the mean of the independent runs.

### Isothermal titration calorimetry

Thermodynamic binding parameters for the binding of nucleotides to the Src kinase domain were obtained through isothermal titration calorimetry as published^63^. All experiments were run in a VP-ITC instrument (Microcal) at 25 °C. Proteins were exchanged into 20 mM Tris (pH 8.0), 250 mM NaCl, 10 mM MgCl_2_ on PD-10 buffer exchange columns (GE Life-science) and diluted to 50–100 μM. Nucleotide stocks (Sigma) were made in buffer to final concentrations between 1 and 3 mM. The heat of binding was measured over the injection of 295 μl of drug in 10-μl steps spaced 300 s apart. Data were fit to a one binding site model using the Origin software package (Microcal). Error bars represent the s.e. of the mean of three independent runs. Representative enthalpograms are shown in Supplementary Fig. 7.

## Additional information

**How to cite this article:** Foda, Z. H. *et al*. A dynamically coupled allosteric network underlies binding cooperativity in Src kinase. *Nat. Commun.* 6:5939 doi: 10.1038/ncomms6939 (2015).

## Supplementary Material

Supplementary Information Supplementary Figures 1-10

## Figures and Tables

**Table 1:** A summary of the key features of the two conformational states visited by the simulation.

	**Pre transition**	**Post transition**
ATP affinity	High	Low
Peptide affinity	Low	High
r.m.s.f. of substrate binding	Low	High
αC-helix	In	Out
Conformation	Active like	Autoinhibited
Asp404 (DFG)	Deprotonated	Protonated
Asp386 (HRD)	Interacts with Arg388	Interacts with Asp404
Lys427	Out of peptide site	In peptide site

r.m.s.f., root mean squared fluctuation.
