# Supplementary material for: A dynamically coupled allosteric network underlies binding cooperativity in Src kinase
Source: Nat Commun. 2015 Jan 20;6:5939. doi: 10.1038/ncomms6939 (PMC4300553; doi:10.1038/ncomms6939)
Supplement: Supplementary Information — Supplementary Figures 1-10 [file ncomms6939-s1.pdf]

Supporting Figures

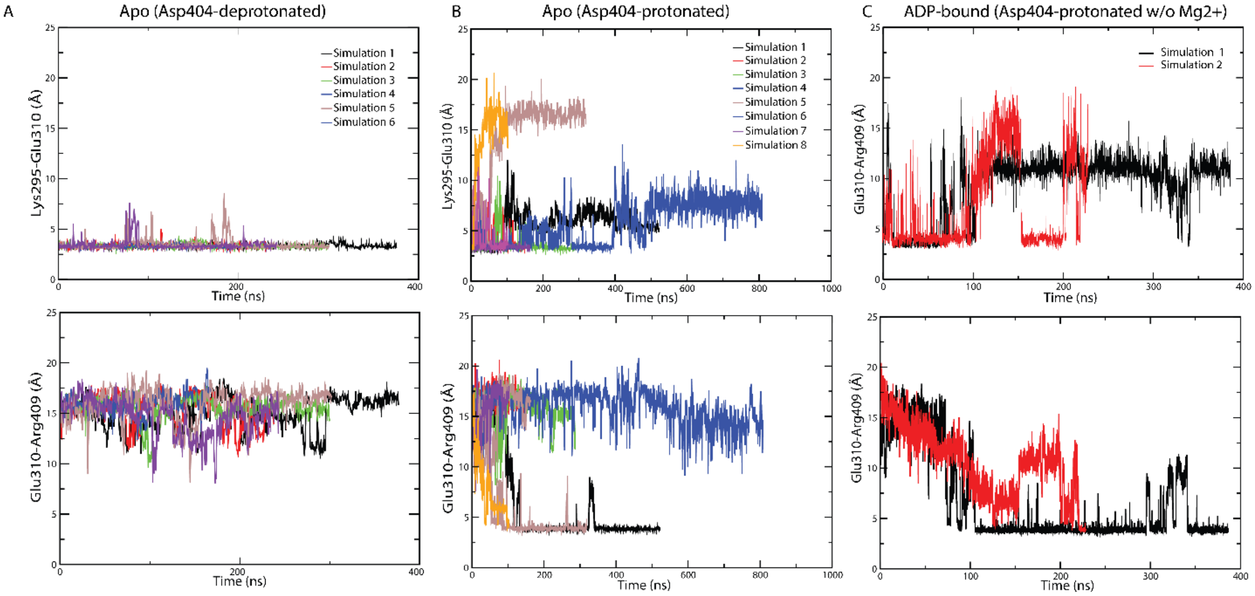

Supplementary Figure 1. Protonation of the DFG aspartate (Asp404) destabilizes the αC-in conformation. Here the disruption of the conserved Lys295-Glu310 salt bridge is used to mark the departure from the αC-in conformation and formation of the Glu310-Arg409 salt bridge is used to mark the αC-out conformation (see Figure 2 in the main text). As shown (A), in the six simulations where Asp404 is not protonated, the Lys295-Glu310 salt bridge remains stable and the Glu310-Arg409 salt bridge never formed, indicative of a stable αC-in (active) conformation. In clear contrast, in three of the eight simulations where Asp404 is protonated (Simulations 1, 5, and 8), the former salt bridge is disrupted and the latter is formed, indicative of a transition to an inactive conformation. (C) Similarly the two salt bridges are shown for the two simulations starting with bound ADP, protonated Asp404 and without Mg^{2+} ions, where theαC-in conformation was not stable.

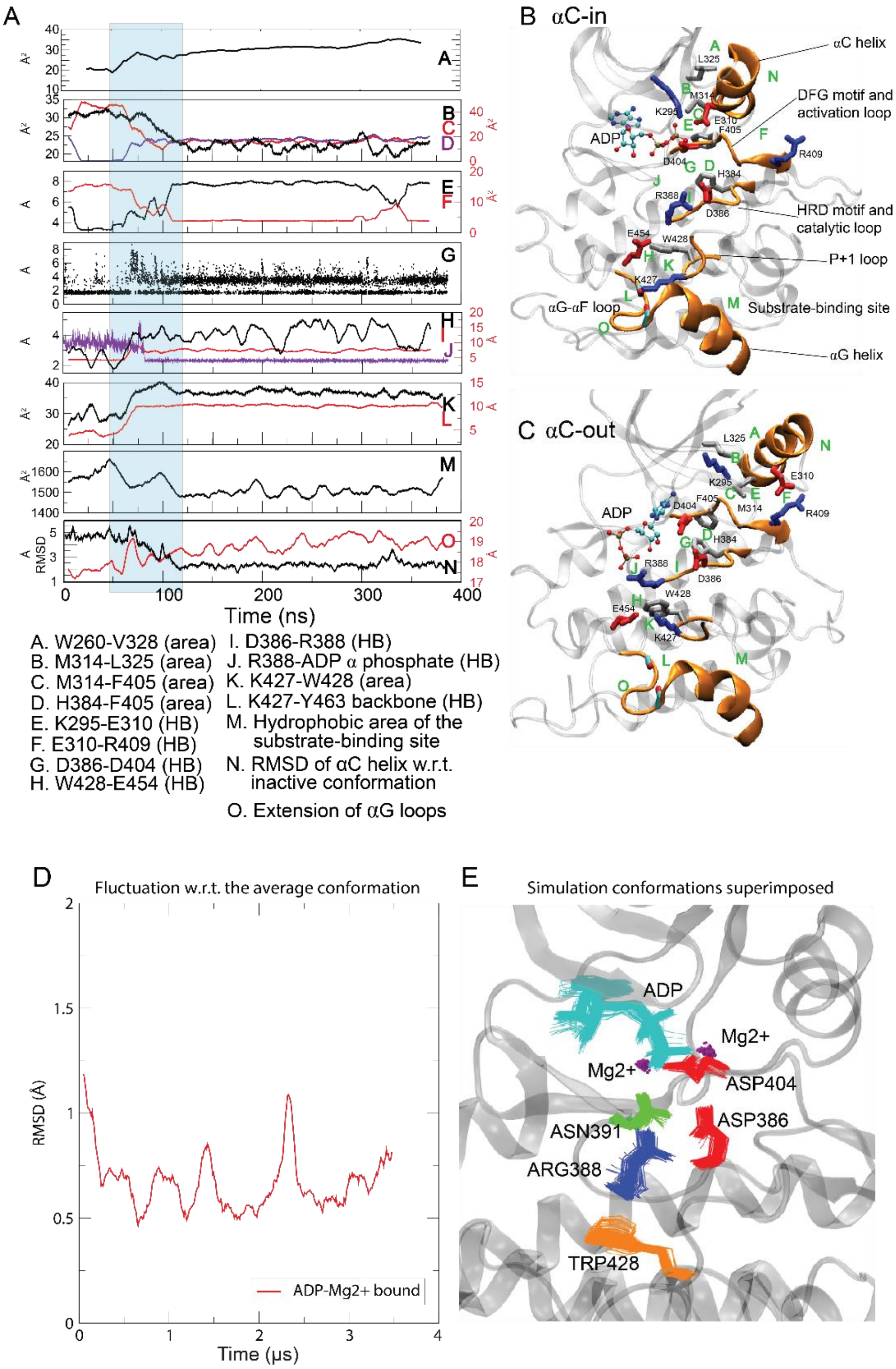

Supplementary Figure 2. The concerted conformational change in a simulation of protonated Src kinase domain bound to ADP in the absence of Mg^{2+} ions.

This figure shows a modification of the simulation presented in Figure 1 (apo Src kinase, Asp404 protonated, Mg^{2+} absent). In the presence of ADP (Src•ADP, Asp404 protonated, Mg^{2+} absent), we observe a concerted conformational change largely involving the same subset of residues as in the absence of ADP. (A) Similar to Figure 1C, selected local structural parameters (distance, contact area, etc.) are plotted as functions of simulation time. (B) The conformation before the conformational change. (C) The conformation after the conformational change. The locations in the kinase domain of the structural parameters plotted in A are marked by their alphabetic labels in B and C. (D) When Mg^{2+} ions are present in the binding site, Asp404, which chelates an Mg^{2+} ion is not protonated, and the conformation of the key residues are highly stable, as reflected by the low RMSD. This is in clear contrast to the cases where both Mg^{2+} ions are absent and Asp404 is protonated. The RMSD is calculated after alignment by all Cα atoms of the kinase domains, using all heavy atoms of the residues shown in (E), the ADP molecule, and the Mg^{2+} ions. (E) The conformations of key residues of the allosteric network that are involved in ADP/ Mg^{2+} binding.

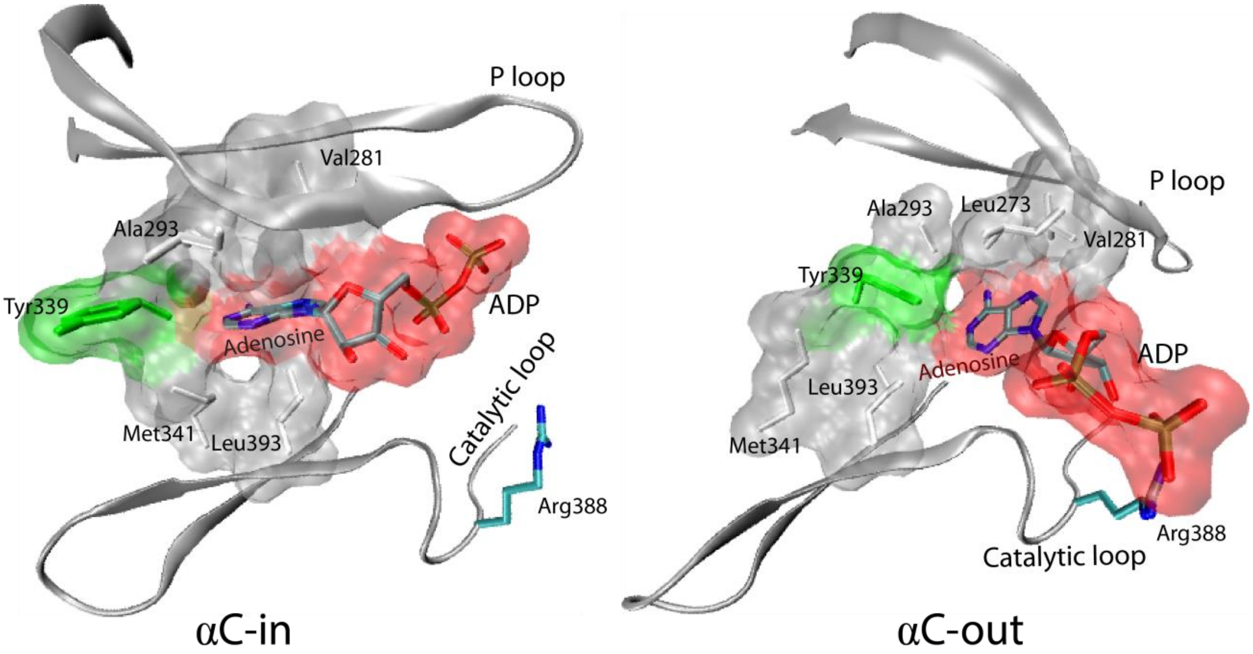

Supplementary Figure 3. Disruption of the catalytic spine at the ATP-binding site. The conformation of the catalytic spine and ADP before and after the concerted conformational change in the simulation of ADP-bound kinase. Hydrophobic residues are rendered in gray and Tyr339 is rendered in green. Arg388 forms an ion pair with the β phosphate of ADP in a possible intermediate conformation for ADP release after the conformational change.

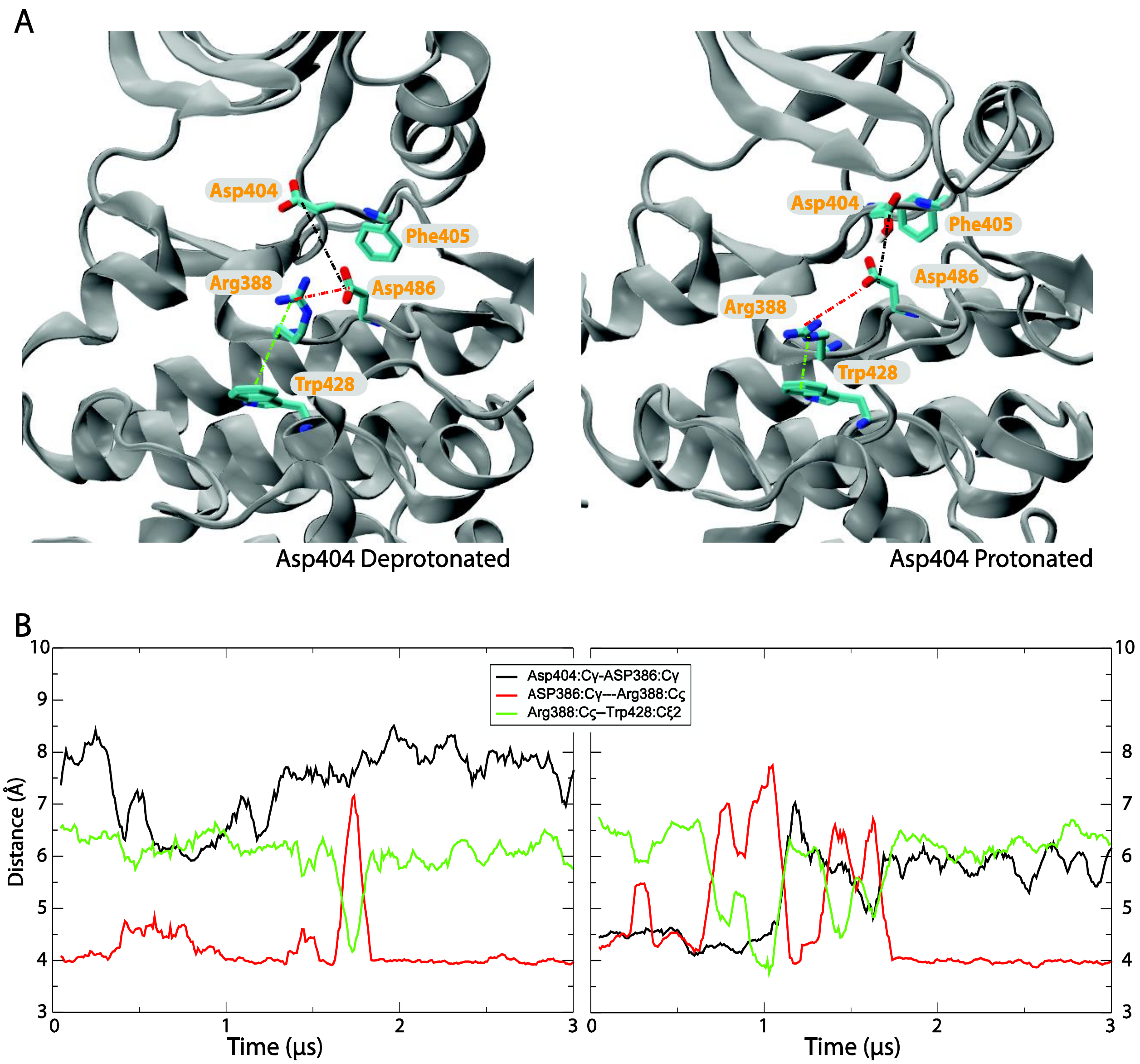

Supplementary Figure 4. Amber simulations: the effect of Asp404 protonation on the conformations of Arg388 and Trp428. (A) Representative conformations of Src kinase with and without Asp404 protonation. As shown, protonated Asp404 tends to form a hydrogen bond with Asp386, while deprotonated Asp404 tends to be more distant. (B) The distance time series suggest that the Asp404-Asp386 interaction is anti-correlated with the latter's salt bridge with Arg388 and separation of Arg388 and Trp428 sidechains.

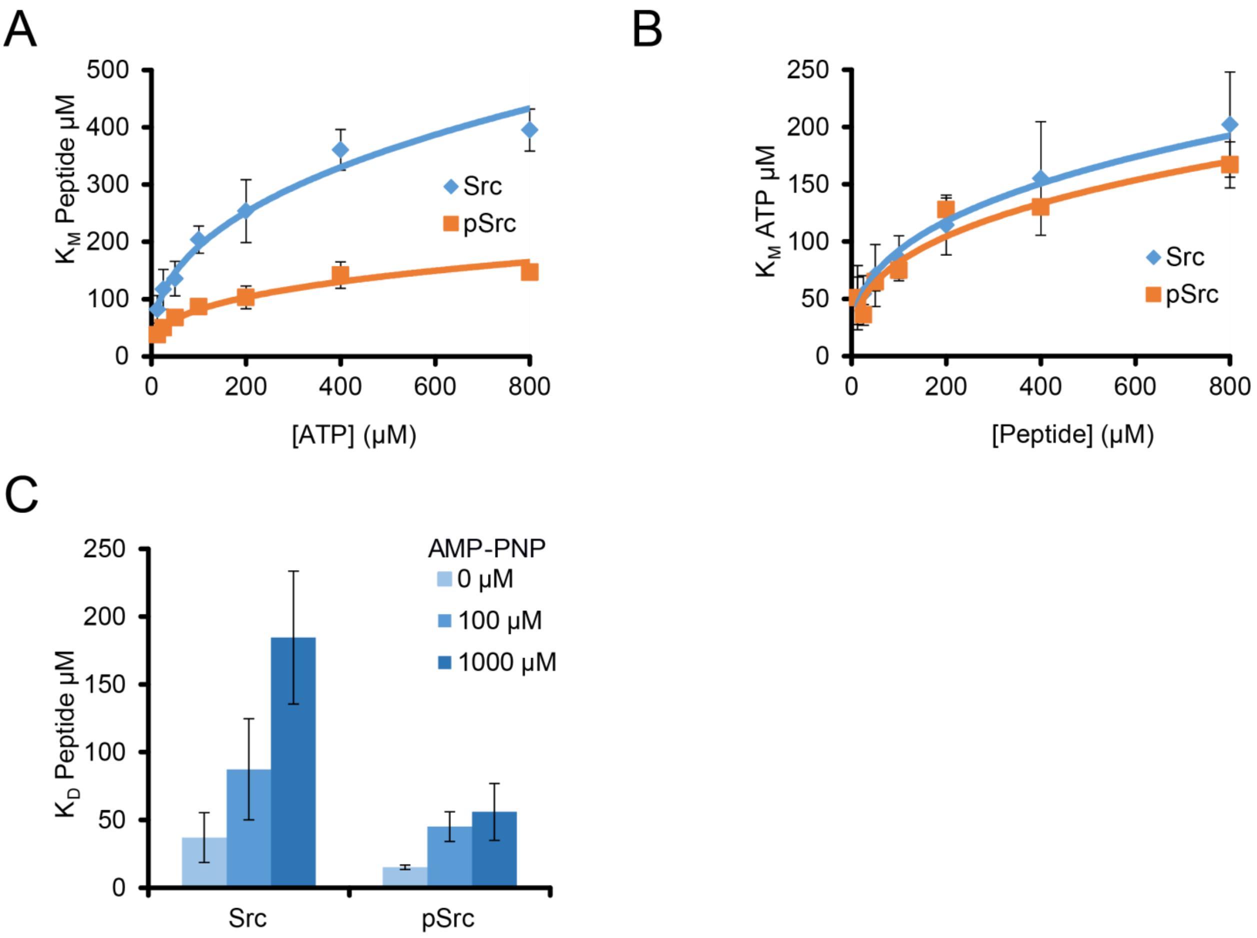

Supplementary Figure 5. Src autophosphorylation increases affinity for substrate peptide. (A) The effect of ATP concentration on substrate K_M for unphosphorylated (blue) and autophosphorylated (orange) Src kinase domain. (B) The effect of peptide concentration on the ATP K_M for unphosphorylated and autophosphorylated Src kinase domain. (C) The effect of AMP-PNP concentration on substrate K_D for unphosphorylated and autophosphorylated Src kinase domain. All experiments were performed in triplicate, and data represent mean values ± s.e.m. For experiments on autophosphorylated Src, the protein sample was pre-incubated with ATP/Mg^{2+} under conditions previously shown (2) to promote near-complete autophophorylation on Tyr416. All experiments were performed in triplicate, and data represent mean values ± s.e.m.

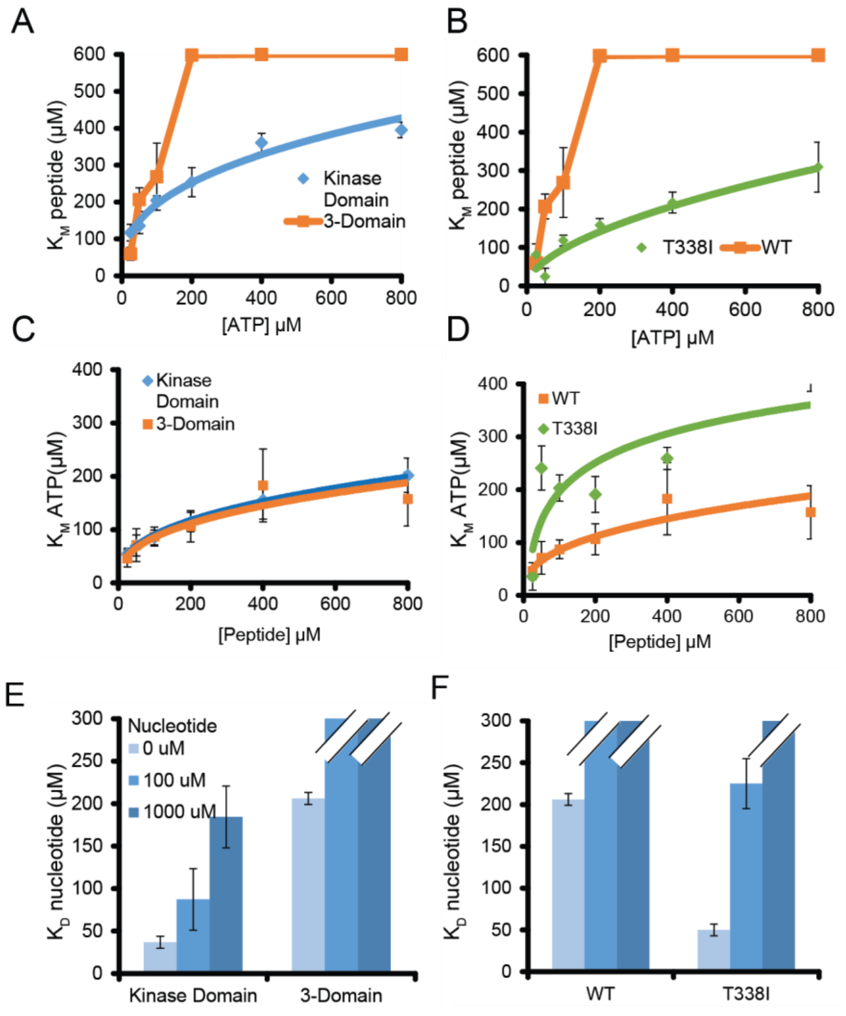

Supplementary Figure 6. Negative cooperativity of ATP and substrate binding in 3-Domain Kinase. (A) The effect of ATP concentration on substrate K_M for Src kinase domain and 3-domain construct. (B) The effect of ATP concentration on substrate K_M for WT and T338I mutant Src 3-domain construct (C) The effect of peptide concentration on ATP K_M for Src kinase domain and 3-domain construct. (D) The effect of substrate concentration on ATP K_M for WT and T338I mutant Src 3-domain construct. (E) Dissociation constants Src kinase domain and 3-domain constructs at increasing AMP-PNP concentration. (F) Dissociation constants for WT and T338I mutant Src 3-domain construct. K_M values were determined in a kinase activity assay. K_D values for the substrate peptides were determined using fluorescence anisotropy at 1 µM labeled peptide. All experiments were performed in triplicate, and data represent mean values ± s.e.m.

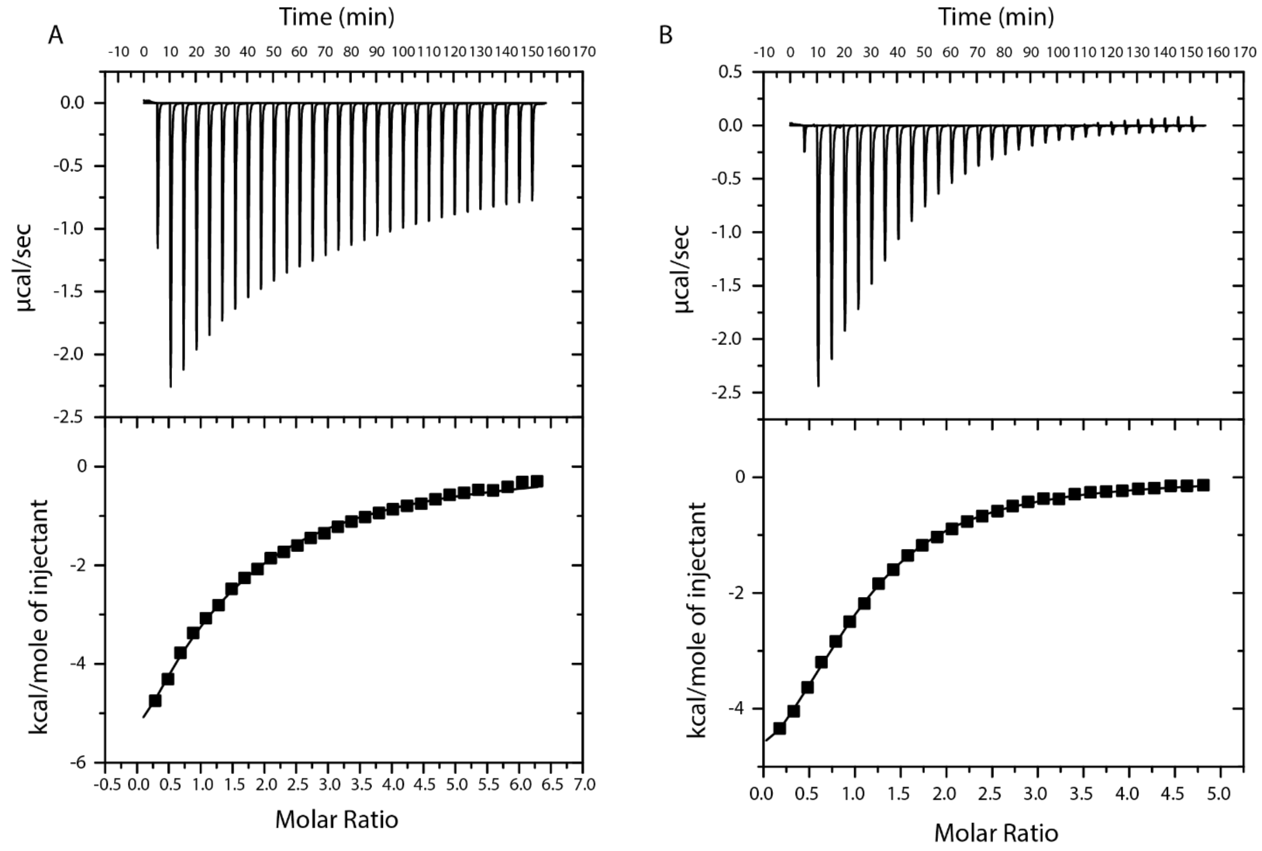

Supplementary Figure 7. Isothermal Titration Calorimetry of Src Binding to AMP-PNP and ADP (A) Sample titration curve for AMP-PNP binding to the Src kinase domain.(B) Sample titration curve of ADP binding to the Src kinase domain. All experiments were run in a VP-ITC instrument (Micro-cal) at 25 °C. Proteins were exchanged into 20 mM Tris (pH 8.0), 250 mM NaCl, 10 mM $MgCl_2$ on PD-10 buffer exchange columns (GE Life-science), and diluted to 50–100 µM.

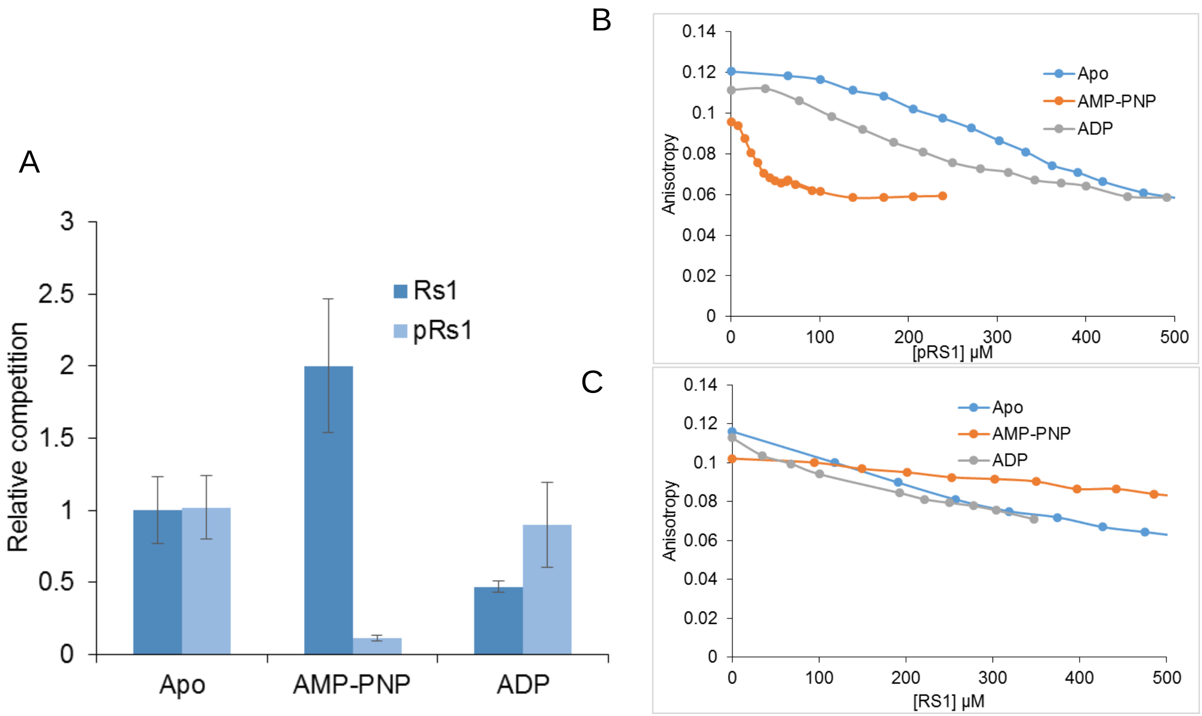

Supplementary Figure 8. Competition experiments examine the effect of nucleotides on phosphorylated substrate binding. A) Concentration at half dissociation of labeled peptide relative to unphosphorylated peptide with apo Src. Error bars represent the 95% confidence intervals of fit. Peptide (B) or phosphorylated peptide (C) was titrated to Src in complex with fluorescently labeled substrate in the absence of nucleotide (blue) or presence of 1mM AMP-PNP (orange) or ADP (grey).

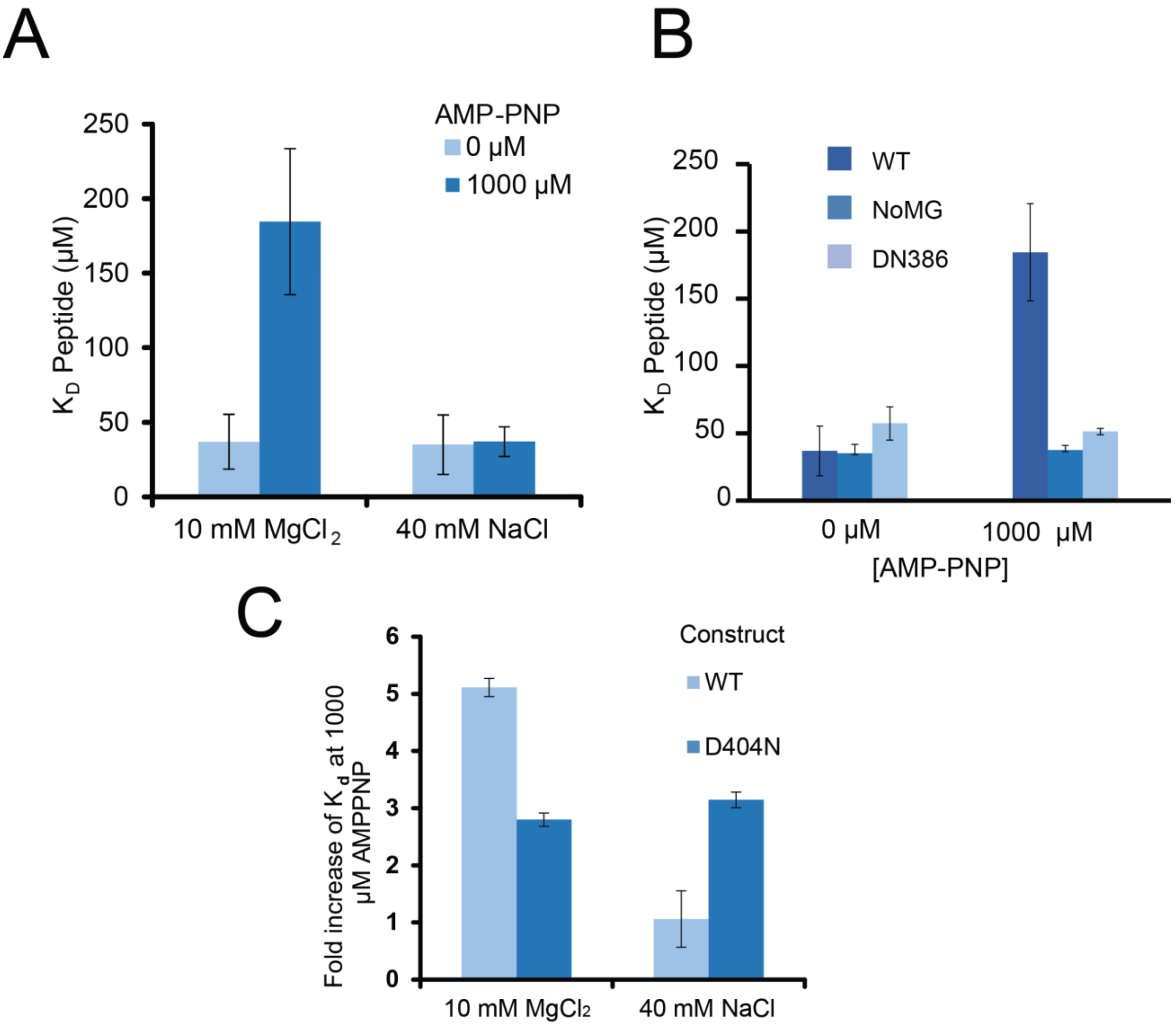

Supplementary Figure 9. Absence of Mg^{2+} disrupts ATP binding and promotes peptide substrate binding. A) The effect of AMP-PNP concentration on substrate K_D in the presence and absence of Mg^{2+}. (Mg^{2+} is required for ATP or AMP-PNP binding in protein kinases [3].) Peptide binding affinity increased and became independent of nucleotide concentration in the absence of Mg^{2+}. B) and C) The effect of AMP-PNP concentration on substrate K_D on D->N mutations in the presence and absence of Mg^{2+} Ionic strength was adjusted to 40 mM with the addition of NaCl. All experiments were performed in triplicate, and data represent mean values ± s.e.m.

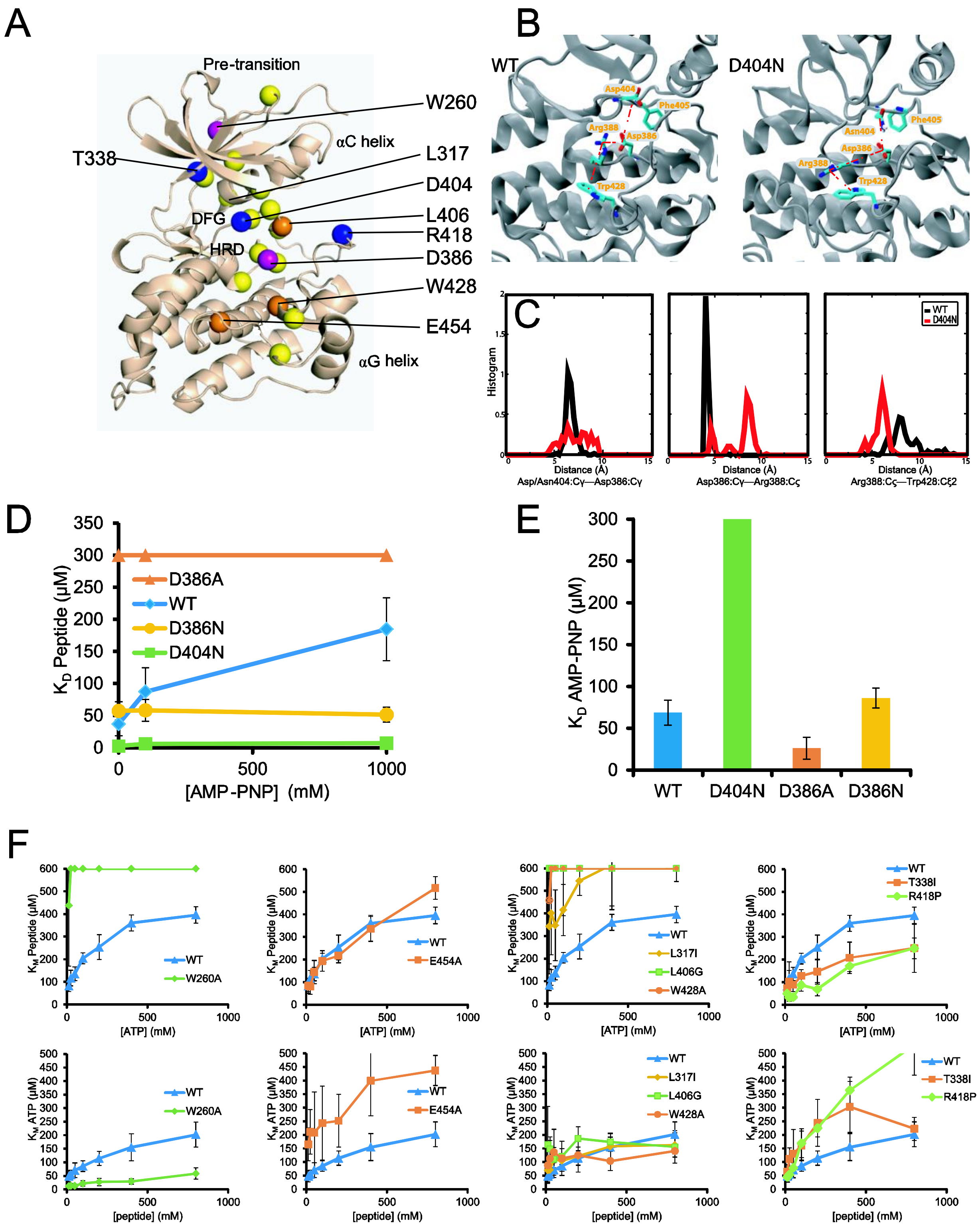

A
Pre-transition
W260
αC helix
T338
L317
D404
DFG
L406
R418
HRD
D386
W428
E454
αG helix
B
WT
D404N
Asp404
Phe405
Arg388
Asp386
Trp428
Asn404
C
Histogram
Distance (Å)
Asp/Asn404:Cγ—Asp386:Cγ
Asp386:Cγ—Arg388:Cς
Arg388:Cς—Trp428:Cξ2
WT
D404N
D
K_D Peptide (μM)
D386A
WT
D386N
D404N
[AMP-PNP] (mM)
E
K_D AMP-PNP (μM)
WT
D404N
D386A
D386N
F
K_M Peptide (μM)
[ATP] (mM)
WT
W260A
E454A
L317I
L406G
W428A
T338I
R418P
K_M ATP (μM)
[peptide] (mM)

Supplementary Figure 10. Mutations to the allosteric network produce biochemical phenotypes. (A) Location of residues involved in the allosteric network (colored beads). (B) Representative conformations of Src wt and D404N. As shown, Asn404 mimics protonated Asp404 by forming hydrogen bonds with Asp386, and affects the conformation of Arg388, Trp428, and other residues involved in the substrate binding. (C) The effects of D404N mutation to three key residue-residue interaction of the putative allosteric network. (D) Dissociation constants of the peptide substrate (RS1) as functions of AMP-PNP concentration. Note that the mutations at D386 disrupt the negative binding cooperativity, and mutation at D404 promotes substrate binding. (E) AMP-PNP dissociation constants of the wild type and D386 or D404 mutants. (F) Substrate-peptide K_M as functions of ATP concentration (upper row) and ATP K_M as functions of peptide concentration (upper row) for Src wild type and mutants. All experiments were performed in triplicate, and data represent mean values ± s.e.m.
